# New progress on the role and mechanism of tau protein in Alzheimer's disease and depression

**DOI:** 10.3389/fneur.2025.1551273

**Published:** 2025-11-28

**Authors:** Yamei Wang, Jinglian Zhou, Qingqing Zhou, Hua Wang

**Affiliations:** 1 Department of Neurology, The First Affiliated Hospital of Yangtze University, Jingzhou First People's Hospital, Jingzhou, China; 2 Department of Neurosurgery, The First Affiliated Hospital of Yangtze University, Jingzhou First People's Hospital, Jingzhou, China

**Keywords:** Alzheimer's disease, tau, β-amyloid, depression, comorbidity

## Abstract

Alzheimer's disease (AD) is an age-related neurodegenerative disease characterized by two major pathological hallmarks: (1) the formation of extracellular β-amyloid (Aβ) plaques; (2) the accumulation of intracellular neurofibrillary tangles (NFTs) composed of phosphorylated tau. The number of NFTs is positively correlated with the severity of AD. However, there are still no effective strategies to treat or slow AD progression. Despite recent approvals of anti-Aβ therapies, their limited clinical benefit has shifted increasing attention toward tau pathology as a parallel driver of AD progression. In recent years, the importance of tau in the pathogenesis of Alzheimer's disease increasingly recognized. The transmission of pathogenic tau proteins in the brain, known as prion-like seeding, is considered a key driving factor for AD. Post-translational modifications of tau—such as hyperphosphorylation, acetylation, glycosylation, ubiquitination, and truncation—promote the onset and progression of Alzheimer's disease. Consequently, tau-targeting therapies have become a major focus in anti-AD research, though most remain at the pre-clinical stage. Furthermore, depression is highly prevalent in AD patients, representing both a potential risk factor and a consequence of the disease. Depression is a risk factor of AD, it is also a consequence of AD. Researchers have found that tau is closely related to depression, not Aβ. This review will focus on tau, tau and AD, post-translational modification of tau, tau targeting strategies, and the role of tau in depression. Since tau pathology not only disrupts synaptic and neuronal networks but also affects limbic and cortical circuits involved in emotion regulation, its dysfunction may underlie depressive symptoms frequently observed in AD. Therefore, understanding tau's neural impact provides a mechanistic bridge between AD pathology and depression.

## 1 Introduction

Dementia is a leading cause of disability among older adults globally, posing a major public health challenge and a substantial economic burden (1, 2). Alzheimer's disease (AD), accounting for 60%−70% of dementia cases, is characterized by a progressive and irreversible decline in cognitive and behavioral functions, notably learning and memory (3, 4). A prevalent and clinically significant comorbidity in AD is depression. While the prevalence of depression in healthy older adults is ~15%−20%, it rises to over 25% in patients with symptomatic AD (5). Critically, depression is considered both a risk factor for developing AD and a common complication following its onset, making their interplay a pressing clinical concern (5).

Alzheimer's disease is characterized by two primary neuropathological hallmarks: amyloid plaques and neurofibrillary tangles (NFTs). Amyloid plaques are extracellular deposits primarily composed of β-amyloid (Aβ) peptides. In contrast, NFTs are intracellular inclusions formed by aggregates of hyperphosphorylated tau protein (6).

AD is clinically divided into early-onset (EOAD, onset before 65) and late-onset (LOAD, onset after 65) subtypes, with LOAD constituting the majority of cases (2). The etiology of LOAD is multifactorial, involving genetic predisposition, aging, environmental influences, and comorbidities such as obesity (7). EOAD accounts for ~10% of cases, with only about 5% of these attributed to pathogenic mutations in genes involved in amyloid processing (e.g., APP, PSEN1, PSEN2) or inheritance of the APOE ε4 allele, a key genetic risk factor for LOAD (2).

The pathogenesis of AD remains incompletely understood, with several hypotheses proposed, including the amyloid cascade, tau, inflammatory, and cholinergic hypotheses. Historically, the amyloid cascade hypothesis dominated the field (6). However, the repeated failure of clinical trials targeting Aβ over the past two decades, despite successfully reducing amyloid load, has challenged its central role and highlighted the need to explore alternative pathways (6).

There is growing evidence that the severity and progression of cognitive deficits in AD correlate more closely with the burden of tau pathology than with amyloid deposition (2, 7, 8). Tau is a microtubule-associated protein abundantly expressed in central nervous system neurons, where it stabilizes microtubules and facilitates axonal transport (9). In AD, hyperphosphorylation induces a conformational change in tau, diminishing its affinity for microtubules (2, 10, 11). The dissociated, hyperphosphorylated tau then self-assembles into soluble aggregates known as tau oligomers, which can further aggregate into insoluble NFTs (12, 13). Notably, accumulating evidence indicates that soluble tau oligomers exhibit markedly higher neurotoxicity compared to NFTs, impairing synaptic function, disrupting mitochondrial activity, and correlating more closely with neuronal loss and cognitive decline (14). The central role of tau aggregation is shared across a spectrum of disorders termed tauopathies, which include AD, progressive supranuclear palsy (PSP), corticobasal degeneration (CBD), Pick's disease, chronic traumatic encephalopathy (CTE), and frontotemporal dementia with Parkinsonism linked to chromosome 17 (FTDP-17) (2, 15).

This review summarizes current knowledge on tau structure and function, its role in AD pathogenesis, and its mechanistic links to depression.

## 2 Pathogenic mechanism of tau protein

### 2.1 Expression and function of tau protein

Tau is a microtubule-associated protein encoded by the microtubule-associated protein tau (MAPT) gene located on chromosome 17q21 (16). It was first identified in 1975 as a key factor promoting microtubule assembly and stabilization in neurons. Tau is predominantly localized in axons, where it stabilizes microtubules essential for axonal growth, maintenance of neuronal polarity, and intracellular transport of organelles and neurotransmitters. In addition to binding microtubules, tau also interacts with actin filaments, contributing to cytoskeletal organization and signal transduction within neurons (17). Through these interactions, tau modulates synaptic plasticity and maintains neuronal connectivity under physiological conditions.

The MAPT gene undergoes alternative splicing of exons 2, 3, and 10, generating six tau isoforms that differ in their N-terminal inserts (0N, 1N, or 2N) and in the number of microtubule-binding repeats (3R or 4R). The “0N3R” designation refers to an isoform containing no N-terminal inserts (0N) and three microtubule-binding repeats (3R), which predominates in the fetal brain, while both 3R and 4R isoforms are expressed at approximately equal levels in the adult brain. Importantly, 4R tau binds microtubules with higher affinity than 3R tau, and mutations affecting exon 10 splicing can disturb the 3R:4R ratio, contributing to the development of tauopathies such as Pick's disease (3R), progressive supranuclear palsy (4R), and corticobasal degeneration (4R).

In Alzheimer's disease (AD), both 3R and 4R tau isoforms aggregate to form neurofibrillary tangles (NFTs). Although NFTs were initially regarded as the main toxic species, increasing evidence suggests that soluble tau oligomers are more neurotoxic, as they impair synaptic plasticity, disturb neuronal communication, and propagate between neurons in a prion-like fashion, thus linking tau's normal physiological role to its pathological transformation that drives neurodegeneration.

### 2.2 Post-translational modification of tau protein

Post-translational modifications (PTMs) regulate tau's interaction with microtubules and affect its physiological and pathological functions. Major PTMs include phosphorylation, O-GlcNAcylation, acetylation, ubiquitination, truncation, nitration, and oxidation (18). Among these, phosphorylation is the most extensively studied. tau contains more than 80 serine/threonine residues and 5 tyrosine residues that serve as potential phosphorylation sites (19). Under normal conditions, a moderate phosphorylation level maintains neuronal plasticity and microtubule dynamics.

However, in Alzheimer's disease (AD), tau becomes hyperphosphorylated, disrupting its microtubule binding and leading to cytoskeletal instability and axonal transport deficits (10). In AD brains, tau phosphorylation levels are two to three times higher than normal (20). Several kinases are implicated in this process. Glycogen synthase kinase 3β (GSK3β) is one of the principal tau kinases, phosphorylating multiple proline-directed sites. Cyclin-dependent kinase 5 (CDK5), when aberrantly activated by p25, induces pathological tau phosphorylation and synaptic dysfunction. c-Jun N-terminal kinase (JNK) links cellular stress to tau pathology, while ERK and p38 MAPK further amplify phosphorylation signals (2). Collectively, these kinases disrupt tau–microtubule interactions, resulting in aggregation and neuronal toxicity. Furthermore, phosphorylation at one site can facilitate phosphorylation at additional residues, indicating a cascade or “spread” effect across the tau molecule (21).

Beyond phosphorylation, other PTMs also modulate tau's stability and aggregation. O-GlcNAcylation competes with phosphorylation at serine/threonine residues: decreased O-GlcNAcylation promotes hyperphosphorylation, while increased modification has protective effects (22). Acetylation, another early pathological event, blocks tau degradation and facilitates its aggregation and intercellular propagation (23). Ubiquitination and truncation contribute to the formation and stabilization of paired helical filaments (PHFs), whereas methylation may suppress aggregation (17). These PTMs are not independent but exhibit significant cross-talk. For example, reduced O-GlcNAcylation can enhance phosphorylation, and acetylation may hinder tau clearance through the ubiquitin–proteasome system. Such interactions collectively determine tau's conformational state and toxicity. Although the precise mechanisms remain incompletely understood, targeting tau PTMs—especially hyperphosphorylation—represents a promising strategy for treating tau-related neurodegenerative diseases.

### 2.3 Pathological transmission of tau protein

Alzheimer's disease (AD) and other noninfectious neurodegenerative disorders associated with protein aggregation share features with prion diseases, particularly the self-propagating spread of misfolded proteins (24). Both tau and Aβ exhibit a characteristic transcellular propagation, where abnormal conformers seed the misfolding of normal proteins. The Braak staging system reflects the predictable spatiotemporal pattern of tau pathology: neurofibrillary tangles (NFTs) first emerge in the entorhinal cortex, then progress to the hippocampus, and eventually involve widespread cortical regions (25).

*In vivo* studies provide strong evidence for this prion-like propagation. Injection of synthetic tau fibrils into the hippocampus or frontal cortex of tau-P301L transgenic mice induced local tau hyperphosphorylation and aggregation, followed by time-dependent spread to anatomically connected regions (26).

Similar findings confirm that misfolded tau acts as a seed, triggering endogenous tau misfolding and pathology propagation across synaptically linked neurons (27). The cellular mechanisms underlying this process involve both release and uptake of tau. Hyperphosphorylated or oligomeric tau can be released into the extracellular space via exocytosis, exosomes, microvesicles, or neuronal death (28). Once outside the cell, tau can be internalized by neighboring neurons or glial cells through endocytosis, macropinocytosis, receptor-mediated uptake, or exosome fusion (28). Notably, low-density lipoprotein receptor-related protein 1 (LRP1) facilitates neuronal uptake of extracellular tau, and LRP1 knockout significantly reduces tau internalization and propagation (29). Exosomes are a particularly important mediator of tau transmission. Pathogenic tau and Aβ species have been detected in brain tissue, cerebrospinal fluid, and plasma of AD patients (30–32). Increased neuronal activity enhances the release of tau-containing exosomes, promoting intercellular spread and subsequent neuronal apoptosis (33). Peeraer et al. (26) discovered that injecting Tau protein filaments into the hippocampus of tau-P301S transgenic mice could lead to selective neuronal loss in the CA1 region of the brain. The use of neutral sphingomyelinase-2 inhibitors can reduce the transmission of Tau protein in adeno-associated virus (AAV) -induced Tau proteinopathy mice and tau-p301S transgenic mouse models (33). In addition to neurons, glial cells play a pivotal role in the pathological propagation of tau. Microglia actively phagocytose tau aggregates and can release tau-loaded exosomes, amplifying the spread of pathology to surrounding neurons (34, 35). Astrocytes can internalize and degrade tau, but excessive accumulation impairs their clearance capacity, contributing to chronic neuroinflammation and neuronal dysfunction (36, 37). Overall, these findings demonstrate that tau propagation involves multi-cellular interactions between neurons and glia, following a prion-like mechanism of transmission. Understanding these pathways provides critical insights for developing therapeutic strategies aimed at blocking tau release, uptake, or exosome-mediated dissemination in AD.

The dominance of the amyloid cascade hypothesis has diminished due to repeated clinical trial failures showing limited cognitive improvement despite Aβ reduction.

## 3 Tau protein and Alzheimer's disease

Tau pathology in AD is characterized by three interrelated processes: loss of microtubule-stabilizing function, gain of toxic function through soluble oligomers, and stereotyped, prion-like propagation across connected brain regions. Among these, soluble tau oligomers correlate more closely with synaptic failure and cognitive decline than insoluble neurofibrillary tangles (NFTs) (38). NFTs, however, remain a robust marker of disease stage.

The phosphorylation state of tau is regulated by a balance between kinases and phosphatases. Among the major kinases, GSK3β and CDK5 phosphorylate tau at multiple disease-associated sites, while JNK and MARK are activated under cellular stress to enhance phosphorylation. In healthy neurons, these kinases are counterbalanced by phosphatases such as PP2A, which dephosphorylates tau and prevents its pathological accumulation. In AD, the activity of kinases is upregulated by upstream signaling pathways (e.g., insulin resistance, oxidative stress, neuroinflammation), whereas PP2A activity is reduced, shifting the balance toward persistent hyperphosphorylation.

In addition to phosphorylation, tau exhibits “prion-like” properties, enabling its pathological spread across brain regions. Misfolded tau oligomers can be released into the extracellular space via exosomes or direct secretion, taken up by neighboring neurons, and then act as templates to misfold normal tau. This self-propagating mechanism parallels prion diseases, where a misfolded protein conformer spreads pathology in a templated manner. In AD, such propagation follows a stereotypical pattern described by Braak staging, beginning in the entorhinal cortex and hippocampus and progressing to widespread cortical involvement (38).

Overall, tau contributes to neurodegeneration in AD through a combination of microtubule destabilization, disruption of intracellular trafficking, toxic oligomer formation, and prion-like propagation across neural circuits. These processes are regulated by complex signaling networks that remain key targets for therapeutic intervention.

### 3.1 The pathological effect of tau protein

Tau protein is usually in an unfolded state and its main physiological role is to maintain the stability of microtubules (15). The tendency of normal tau proteins to misfold, aggregate and accumulate is very low (10). However, abnormal modification of tau protein can cause its “prion-like” diffusion, causing abnormal folding and aggregation of tau protein, resulting in tau disease. Although phosphorylation of tau protein plays an important role in physiological conditions, tau hyperphosphorylation results in conformational change and aggregation of tau protein. Abnormally aggregated tau proteins form NETs, which cause damage to nerve cells (10).

Prior to the formation of NETs, hyperphosphorylated tau proteins at the C-terminus assemble to form PHF. tau protein aggregation in PHF conformation leads to impaired axonal transport of neuronal cells, resulting in microtubule instability. The phosphorylation level of tau protein in AD patients is 4 times that of normal people (10). In addition, misfolded tau proteins lose their ability to maintain microtubule stability while increasing the aggregation effect, which is considered a potential neurotoxin. Ultimately, synaptic remodeling and axonal transport caused by impaired tau-microtubule function ultimately lead to cognitive impairment in patients. Therefore, abnormal phosphorylation of tau protein is considered to be the main pathological change of tau protein, which is closely related to the occurrence and development of AD (11).

### 3.2 Regulation of tau protein

Tau protein can be regulated after translation by over-modification, including phosphorylation, acetylation, glycosylation, ubiquitination, and truncation. The functional characteristics of tau proteins depend on and are regulated by post-translational modifications (39). Phosphorylation and dephosphorylation are the most common modifications of tau protein and are regulated by specific protein kinases and phosphatases (40–42). Instead of listing each kinase and phosphatase individually, the major regulatory enzymes are summarized in Table 1 to enhance readability.

**Table 1:** Key enzymes involved in tau phosphorylation and dephosphorylation.

**Category**	**Representative enzymes**	**Functional consequence**
Kinases	GSK-3, CDK5, MARK, MAPK, PKA	Promote tau hyperphosphorylation and aggregation
Phosphatases	PP2A, PP1, PP5	Reverse tau phosphorylation, restore microtubule stability

#### 3.2.1 Phosphorylation of tau protein

Protein kinases (PK) responsible for tau protein phosphorylation can be divided into three main categories, namely, proline-directed protein kinases, PDPK, non-proline directed protein kinase (non-PDPK) and tyrosine protein kinases (TPK) (10). Glycogen synthase kinase-3 (GSK-3), cyclin-dependent kinase 5 (cdk5), C-Jun amino terminal kinase (JNK) belongs to the PDPK family of kinases (10). casein kinase 1 (CK1), dual specificity tyrosine-phosphorylation-regulated kinase 1A (Dyrk1A), adenosine-monophosphate activated protein kinase (AMPK), microtubule-affinity regulating kinases (MARKs), cyclic adenosine monophosphate (cAMP) -dependent PKA, tau protein kinase I (TPKI) and tau protein kinase II (TPKII) belong to the non-PDPK family of kinases (10, 40). In addition, protein phosphatase 2B (PP2B), protein phosphatase 2A (PP2A), protein phosphatase 1 (PP1), protein phosphatase 5 (PP5), calcyclin binding protein and Siah-1 interacting protein, CacyBP/SIP proteins and tissue-nonspecific alkaline phosphatases (TNAP) are also different types of protein phosphatases (PPs) (10).

GSK-3, cdk5 and JNK are serine/threonine specific kinases whose activity is regulated by phosphorylation (10). The phosphorylation sites of tau protein are regulated by different phosphorylated kinases. GSK-3 is activated when phosphorylated at either tyrosine 279 (GSK-3α) or tyrosine 216 (GSK-3β), while cdk5 is phosphorylated at tyrosine 15 (10). Non-pdpks also phosphorylate tau proteins at different sites. For example, when tau's Ser202/Thr205 and Ser396/Ser404 sites are phosphorylated by CK1, tau's microtubule binding affinity changes. In addition, some kinases phosphorylate tau at multiple sites, such as Dyrk1A phosphorylates tau at Thr212, Ser202, and Ser404 (10).

In tau, nearly 50% of phosphorylation sites are followed by proline residues (10). PDPKs play an important role in tau protein phosphorylation and have been shown to be associated with the onset and development of neurodegenerative diseases such as Pick's disease, progressive supranuclear palsy, and AD (10). In addition, it has been demonstrated in transgenic mouse models that the upregulation of GSK3 and CDK-5 regulatory protein p25 is associated with the hyperphosphorylation of tau protein and can cause spatial learning deficits in mice (43–45). On the other hand, non-proline sites can be phosphorylated by non-PDPKs, such as PKA and Mark. Under physiological and pathological conditions, tau protein polymerization is regulated by both PDPK and non-PDPK.

#### 3.2.2 Acetylation of tau proteins

There are more than 20 lysine residues on tau protein, which are located in the C-terminal microtubule-binding repeats (MTBRs) and flank regions of tau protein. It is acetylated by CREB-binding protein (CBP) or histone acetyltransferase p300, and deacetylated by histone deacetylase 6 (HDAC6) or sirtuin 1 (SIRT1) (9). The acetylation of tau protein can block the degradation of tau protein, prevent tau protein and microtubule tuberculosis, and promote tau protein aggregation (46). The study found that in brains with tau disease, the level of acetylation of tau protein increased (9). In a mouse model of AD, acetyltransferase p300 was increased in the mouse brain (46). In the AD brain, levels of the deacetylase SIRT1 decreased (47). *In vitro* experiments, the expression of SIRT1 was reduced in neurons treated with Aβ (48), this leads to persistent acetylation of tau proteins. Thus, either increased acetylation or decreased deacetylation can lead to a pathological increase in tau acetylation in AD (46).

#### 3.2.3 Glycosylation of tau protein

O-GlcNAcylation, also known as O-GlcNA glycosylation, is a modification of nucleoplasmic proteins that binds primarily to the serine or threonine residues of the hydroxyl group at the monosaccharide β-n-acetylgluconediamine (GlcNAc) (49). tau protein can also undergo O-GlcNA glycosylation (50, 51). O-GlcNA glycosylation is mainly accomplished by O-GlcNAc transferase (OGT), which is dissociated by O-GlcNAc hydrolase (OGA). The glycosylation level of O-GlcNA is determined by the activity of OGT and OGA and the content of UDP-GlcNAc. Site-specific phosphorylation and O-GlcNA glycosylation of tau proteins have competing effects (9). The decrease of O-GlcNA glycosylation of tau protein promotes abnormal hyperphosphorylation of tau protein and NFTs formation. In AD brain, Ca2+ overload can cause excessive activation of Calpain I, leading to hydrolysis of neuron-specific glucose transporter 3 (GLUT3), impelling glucose uptake by neurons. This results in a decrease in the glycosylation of tau protein O-GlcNA (9). Therefore, improving glucose uptake in the brain is one of the potential targets for preventing and treating AD.

#### 3.2.4 Ubiquitination of tau protein

Ubiquitination is a post-translational modification with multiple functions (52). Ubiquitination can degrade proteins through the ubiquitin-proteasome system (UPS), or it can degrade proteins independently of the proteasome. Tau protein has some lysine residues that can be ubiquitized by ubiquitin ligase modification (E3 ligase) (9). Neuritis plaques and tau ubiquitination modification of NFTs were found in AD brains. Ubiquitination of tau protein is also seen in Parkinson's disease and Pick's disease (9).

During the initial phase of AD, the hippocampal region of the brain shows decreased proteasome activity. Proteasome activity decreased with the aggregation of tau protein and Aβ. In AD brain, soluble PHFs ubiquitination occurs mainly at K254/311/353 residues of MTBRs. What usually occurs in PHFs is monoubiquitination of tau proteins, not polyubiquitination, which is insufficient to drive UPS-regulated proteolysis (9). Therefore, tau polyubiquitination and protein degradation are promising therapeutic strategies for AD.

#### 3.2.5 The truncation of tau protein

Tau protein can be abnormally truncated at some sites, and the enzymes that catalyze this process mainly include calpsin, caspase, aspartic endopeptidases asparaginyl endopeptidases (AEPs) and cathepsin (9). At least three specific tau cleavage sites (N368, E391, and D421) in the NFTs of the AD brain have been shown to correlate with the progression of AD (9). The tau protein in NFTs is mainly C-terminal truncated, and half of the tau protein in cerebrospinal fluid (CSF) does not contain the C-terminal portion of MTBR.AD brain contains a large number of high molecular weight tau protein aggregates (HMW-tau) resistant to sodium dodecyl sulfate (SDS) and reducing agents, and there is no N-terminal region. Truncated tau proteins are more likely to aggregate than full-length tau proteins. Recent studies have found that tau truncation can regulate protein phosphorylation at specific sites and promote tau protein aggregation (9). Of all the tau truncated types, tau151-391 has the strongest aggregation and can cause the most severe pathological disorders. Tau truncation can also lead to mitochondrial dysfunction and synaptic defects (9). Therefore, tau truncation plays an important role in the pathogenesis of tau disease and is a potential target for treatment.

### 3.3 Interacting proteins that enhance tau's pathogenicity

Phosphorylation and dephosphorylation of tau protein are important factors in the occurrence and development of AD (10). The study found that tau's pathogenic effect can be enhanced by some proteins. Amyloid-β, Pin1, Fyn kinase, heat shock protein, FKBP51 and FKBP52 immunotropins, α-synuclein, PACSIN1 can directly affect tau protein phosphorylation and dephosphorylation (10). Thus, in addition to post-translational modifications, protein interactions can also regulate tau proteins (10). Taken together, phosphorylation, acetylation, and loss of O-GlcNAcylation converge on the same downstream events: detachment of tau from microtubules, increase of the soluble tau pool, and facilitation of seed-competent species. This enlarged cytosolic pool provides the substrate for prion-like propagation.

### 3.4 The interaction of Aβ and tau proteins

Through direct or indirect interaction with tau protein, Aβ can promote tau protein aggregation, hyperphosphorylation and mis-translocation (10). Studies have shown that Aβ can promote the occurrence and development of tau protein disease by activating specific kinases (53–56). *In vitro* studies have shown that Aβ can cause changes in tau protein, including up-regulation of its phosphorylation and promotion of its translocation to cytoplasm and dendrites (57). In addition, misfolded Aβ also upregulates the planting and aggregation of tau proteins.

Aβ up-regulates tau phosphorylation by activating GSK-3β. In addition, blocking two major tau kinases (cdk5 and GSK-3β) had A protective effect against Aβ-induced neuronal damage, suggesting that tau protein and its phosphorylation play an important role in Aβ-mediated neuronal toxicity. In addition, Fyn kinase plays A key role in the function of synapses and also plays an important role in Aβ-mediated neuronal toxicity. Overexpression of Fyn in AβAPP transgenic mouse models accelerates cognitive impairment and synaptic damage (58, 59). Aβ oligomers bind to the PrPC/mGluR5 receptor complex on the surface of neurons and can lead to A series of biochemical changes in neurons, including the activation of Fyn, which promotes Aβ-induced synaptic toxicity and pathological changes of tau proteins. Fyn also causes the phosphorylation of NMDA receptor subunit−2, which affects the stability of NMDA interactions with PSD95 (the scaffold protein of the dendritic spines of neurons), thereby enhancing the toxicity of Aβ (10).

In the occurrence and development of AD, Aβ and tau proteins are recognized to have A synergistic effect and are key proteins in the pathogenesis of AD. It was found that the accumulation of Aβ was earlier than the pathological change of tau protein, and Aβ had A regulatory effect on tau protein, resulting in A pathological cascade reaction. Injection of synthetic unaggregated Aβ in animal models resulted in pathological aggregation and diffusion of tau protein (10). In addition, recent studies have reported that tau protein aggregation and planting in the hippocampus and parahippocampal gyrus with Aβ plaques are 2–3 times higher than those without Aβ plaques (12, 54, 57, 60). Recently, Mattsson-Carlgren et al. detected upregulation of P-tau, specifically P-tau217, in the cerebrospinal fluid of patients with AD. The level of P-tau in cerebrospinal fluid is closely related to Aβ and tau accumulation (61). In addition, Aβ binds to microglia and astrocytes, activating inflammatory pathways that lead to pathological deposition of tau proteins.

### 3.5 Potential therapeutic measures targeting tau protein

To date, there is no effective treatment for AD. Anti-aβ drugs showed no significant benefit in improving AD symptoms. The development of new drugs for other targets is therefore crucial. Tau protein plays an important role in the occurrence and development of AD and is a potential important target for anti-AD therapy. Currently, drugs targeting tau are in the early stages of development, but have shown considerable translational potential. Tau-directed therapies represent a promising yet challenging frontier in AD treatment. Most interventions remain in the preclinical or early-clinical stage, and translation to human benefit is still limited. Future work should focus on combination strategies addressing both Aβ and tau pathology, improving drug delivery across the blood–brain barrier, and developing biomarkers for early detection and treatment monitoring.

#### 3.5.1 Inhibition of tau protein expression

Several tau-lowering approaches have now entered early clinical development, including antisense oligonucleotides (ASOs) targeting MAPT mRNA (e.g., BIIB080/IONIS-MAPTRx, phase 1b/2a) and tau-directed monoclonal antibodies for PSP and AD, indicating that tau reduction is clinically feasible, although long-term safety and efficacy still remain to be established. tau protein, as A key molecule in the pathogenesis of AD, is directly toxic to cells and mediates Aβ toxicity. Therefore, inhibition of tau protein expression is a potential therapeutic measure for AD. In A mouse model of AD with Aβ-induced cognitive impairment, reducing endogenous tau levels was protective against behavioral abnormalities in the mice (9). Knock-out (KO) of tau gene in mice has almost no obvious side effects, which may be because other related proteins interact with microtubules in place of tau protein to a certain extent (62). If the level of tau monomer in the cell is reduced, the balance of tau aggregation-dissociation will tend toward dissociation, and tau aggregation will decrease (63). Antisense oligonucleotides (ASOs) or small interfering Rnas (SiRnas) can reduce tau expression (9). In both cellular and animal models, siRNA has been shown to reduce the pathological changes and associated functional impairment of tau protein (18). At present, siRNA has only been clinically studied in diseases such as tumors, and there are no ongoing clinical trials in AD and other tau protein diseases. Several microrRNA, including miR-106b, miR-125b, miR-132/122, and miR-219, regulate tau protein expression and phosphorylation (9). ASOs used to be a common experimental method, but is now used less frequently due to its side effects. In recent years, ASOs has been found to reduce the progression of spinal muscular atrophy, and its role in tau protein disease has been re-examined (64, 65). However, whether inhibition of tau protein expression is beneficial in AD remains to be further investigated.

#### 3.5.2 Inhibit tau protein phosphorylation

The hyperphosphorylation of tau protein easily leads to the formation of tau protein aggregates and causes the pathological transmission of tau protein. Therefore, inhibition of tau protein hyperphosphorylation is a potential therapeutic measure for AD. Important kinases in tau phosphorylation include cdk5, GSK-3β, ERK, and Dyrk1A (9). Lithium chloride (LiCl) is a specific inhibitor of GSK-3β. K252a is a nonspecific inhibitor of cdk5, ERK1, and GSK-3, both of which reduce tau hyperphosphorylation in mouse AD models (10). Other small molecules targeted to inhibit GSK-3β include SRN-003-556, SHIR-98014 and SB 216763, all of which are currently in preclinical stages (9). Because tau kinase activity is closely related to tau protein pathology, it is a potential target for the treatment of AD.

#### 3.5.3 Inhibit tau protein aggregation

Some post-translational modifications can reduce the binding ability of microtubules to tau protein and enhance its dissociation from microtubules. The above effects can lead to increased levels of tau within the cell, which increases the probability of interaction with tau proteins and ultimately leads to tau protein aggregation. Tau oligomers are highly neurotoxic and can lead to neurodegeneration of AD. Tau aggregation inhibitors (TAIs), which aim to prevent the pathological transmission of tau protein “prion-like,” are a promising strategy for targeting tau protein.

To date, most TAIs are derivatives of methylene blue (9). TAIs has the ability to destroy tau tangles and tau filaments, preventing cognitive deficits in mice genetically modified for tau (66). Methylthioninium chloride (MTC) is a Drug approved by The US Food and Drug Administration (FDA) for the treatment of methemoglobinemia, And was reintroduced to treat or slow AD progression and other tau diseases (10). Although MTC and other methylene derivatives can reverse PHF proteolysis, block tau protein aggregation, and do not interfere with the interaction between tau and microtubules *in vitro*, the effect of such drugs *in vivo* studies is inconsistent with *in vitro* experiments, and clinical benefits are limited (67).

#### 3.5.4 Inhibition of tau intracellular transport

Aggregates of tau proteins, as well as misfolded tau proteins, can travel from cell to cell in a “prion-like” manner and are released outside the cell, causing pathological changes in tau to spread to different areas of the brain. Intervening in the spread of tau protein is a potential direction for treating tau disease and other neurodegenerative diseases. A large number of studies have shown that blocking tau protein transfer between cells can delay the pathological changes of tau protein in AD patients. Due to the neurotoxicity of tau protein, stopping the pathological transmission of tau protein and reducing the accumulation of tau protein can alleviate and delay the progression of tau-related diseases. There are currently three pathways that can block or reduce tau transfer in neurons, including blocking tau release, inhibiting tau uptake, and reducing tau oligopolization and extracellular tau levels. When tau release is blocked, extracellular tau levels decline, resulting in a decrease in tau uptake by neighboring neurons. Because microglia can both clear and relay tau via exosomes, strategies that modulate microglial exosome release (e.g., nSMase2 inhibition) are being explored as adjunctive tau-spread blockers (9).

#### 3.5.5 Stable microtubule

Impairment of normal tau protein function leads to abnormalities in axon transport and microtubule assembly. The pathological tau protein separates from the microtubule, causing microtubule rupture. Thus, stabilization of microtubules is considered a remedy for tau-induced neurotoxicity. Targeting microtubule stabilization is also a potential treatment strategy for tau disease. Paclitaxel-derived Ebomycin is a small molecule that stabilizes microtubules and can cross the blood-brain barrier. Studies on transgenic mice found that Ebomycin can reduce the loss of neurons in the hippocampus, pathological changes of tau protein, reduce the number of abnormal axons, and increase the number of microtubules, thereby restoring spatial memory loss in mice (68–70). Abeotaxane and davunetide are recently discovered microtubule stabilizers, but their effectiveness in animal models and clinical trials has been inconsistent (63, 68). Other microtubule stabilizers, such as TPI287 and NAP, are in phase I/II clinical trials (63). The clinical effectiveness of drugs targeting microtubule stabilization needs to be further investigated.

#### 3.5.6 Tau immunotherapy

The most promising currently targeted treatment for tau may be tau immunotherapy. Tau immunotherapy can reduce tau phosphorylation and pathological accumulation through passive immunization or active immunization by injecting p-tau antibodies, thereby alleviating clinical symptoms of AD patients.

An antibody to tau (BIIB092), which primarily diagnoses the n-terminal region of tau, was well-tolerated in the Phase 1b PSP clinical trial (71). However, the Phase II trial of the study failed and was discontinued (9). Many other tau antibodies, including those obtained from the serum of AD patients, have shown potential efficacy against tau disease in some preclinical studies (63). UCB0107 was well-tolerated and had an acceptable safety profile in patients with PSP (9). Some antibodies against p-tau (RO6926496, RO7105705), antibodies against tau protein fragments (BMS-986168, C2N 8E12), antibodies against tau protein conformation (antibodies specific to tau oligomers), And an antibody against total tau protein (ABBV-8E12) is currently in clinical trials (72, 73). The above-mentioned immunotherapy targeting tau does not eliminate the neurodegenerative changes already present in AD patients, but inhibits the spread of pathogenic tau to unaffected brain regions, thereby delaying or even stopping the pathological changes of tau (9). Recent studies have shown that tau protein antibody 43D (targeting tau protein 6-18) can block the cultivation and diffusion of hyperphosphorylated tau protein in AD mice, which is a potential transformational strategy for the treatment of AD (74–76).

#### 3.5.7 Future perspectives on tau-centric therapeutics

Tau-centric therapeutics are emerging as a promising avenue in the treatment of AD and tau-related neuropsychiatric disorders. Compared with anti-Aβ approaches, tau-targeted strategies may more directly influence disease progression, as tau pathology shows a closer correlation with cognitive decline. Current therapeutic pipelines include antisense oligonucleotides, small-molecule inhibitors of tau phosphorylation or aggregation, microtubule-stabilizing agents, and active or passive immunotherapies. Despite encouraging preclinical results, most of these candidates remain in early clinical stages, and translation into effective therapies is still challenging. Key obstacles include achieving blood–brain barrier penetration, reducing off-target effects, and demonstrating long-term safety in elderly patients. Nevertheless, the rapid development of novel biologics, advanced delivery systems, and biomarker-guided clinical trials provides optimism that tau-centric approaches will eventually complement or surpass traditional amyloid-based strategies. Future research should emphasize combinational regimens that integrate tau-directed interventions with other disease-modifying therapies to maximize clinical benefit.

## 4 Tau protein and depression

### 4.1 Depression and AD comorbidities

In addition to its pivotal role in AD, tau pathology has also been linked to neuropsychiatric symptoms, especially depression. While amyloid pathology shows limited correlation with mood disturbances, tau accumulation and hyperphosphorylation appear to contribute more directly to depressive symptoms. This highlights the importance of considering tau not only as a marker of cognitive decline but also as a mediator of emotional and behavioral changes in patients. Depression, characterized by low mood, insomnia, altered appetite, feelings of worthlessness and guilt, occurs in at least 4.4% of the population (77). Risk factors for depression include age, stressful life events, endocrine disorders, tumors, drug side effects, socioeconomic status, genes and genetics (78). Depression is very common in older adults and has been linked to an increased risk of cognitive impairment and dementia in older adults (79). Tang et al. (80) reported that among older adults, the prevalence of depression is 20%.

Cognitive impairment is a common concomitant symptom of long-term depression in older adults. Older adults with depression are more likely to have cognitive impairment than those without depression. The cognitive dysfunction of the elderly is mainly manifested as the decline of information processing speed and executive ability (79). A recent case-control study showed that 26.6% of elderly patients with depression were associated with cognitive impairment (81). Ismail et al. (82) reported in a meta-analysis that 32% of patients with mild cognitive impairment had depression. Depression is thought to be an important factor in promoting the progression of mild cognitive impairment to AD (83). Studies have found that depression is not only a risk factor for AD, but also one of the complications and early indications of AD (79, 82). It has been reported that the incidence of depression in AD patients ranges from 14.8 to 40% (84). Therefore, depression and AD comorbidity are common clinical manifestations.

**Figure 1:**
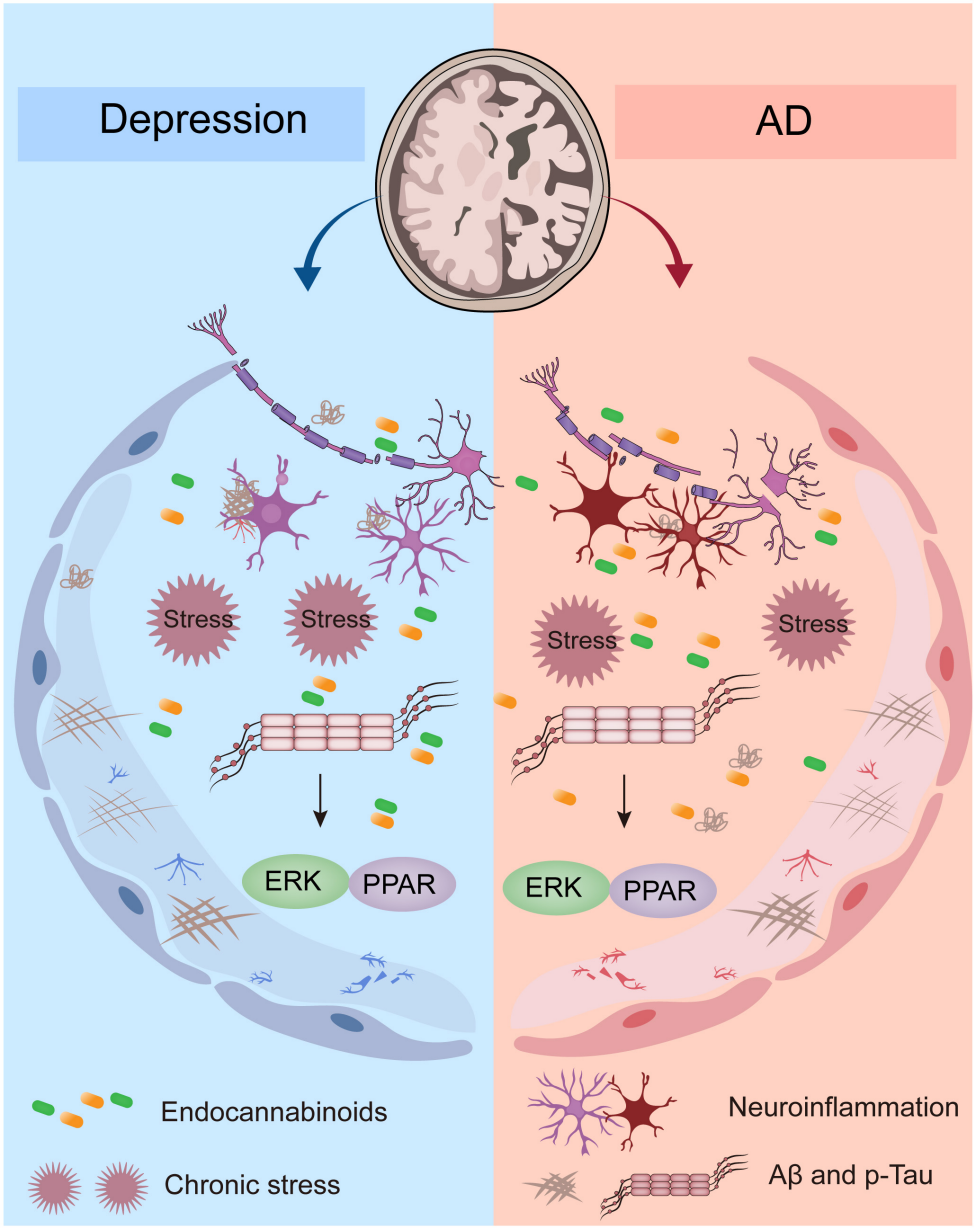
Possible mechanisms of comorbidity between depression and AD.

Figure 1 illustrates the possible mechanisms of depression and AD comorbidity: (1) disturbance of the endocannabinoid system (ECS): the ECS system includes cannabinoid receptors (CBR), endocannabinoids, and proteins involved in the production, transport, and metabolism of both. The dysregulation of ECS is associated with depression and AD (78). (2) Chronic stress: chronic stress is also one of the possible factors of depression leading to AD (85). Chronic stress can lead to increased production of glucocorticoids, activation of the hypothalamus-pituitary-adrenal gland Axis (HPA), and activation of inflammation, resulting in brain damage, especially in the hippocampus, resulting in cognitive impairment. In addition, elevated glucocorticoids were associated with abnormal deposition of tau protein (78). (3) Neurogenic inflammation: neurogenic inflammation is associated with an increase in inflammatory factors in the blood and cerebrospinal fluid, such as IL-6, IL-1β, IFN-α, TNF-α, etc. Several studies have suggested that neurogenic inflammation, defined as inflammatory processes triggered by abnormal neuronal activation and release of pro-inflammatory mediators, may contribute to neuronal dysfunction and mood disturbances (78). (4) Abnormal Aβ and tau protein phosphorylation: depression and depression-like symptoms are associated with abnormal Aβ deposition. In addition, depression was significantly associated with abnormal phosphorylation of tau protein. Abnormal phosphorylation of Aβ and tau proteins is an important pathological feature of AD. To improve readability, the major epidemiological data of Alzheimer's disease worldwide and by region are summarized in Table 2.

**Table 2:** Epidemiology of Alzheimer's disease (AD).

**Region/population**	**Estimated prevalence or number of cases**	**Key notes**
Worldwide (all dementias)	>55 million (2020)—Alzheimer's disease international (ADI)	Includes AD and other types of dementia
Global AD (preclinical + prodromal + dementia stages)	≈416 million individuals aged ≥50 years (≈22% of this population; GBD 2021 data)	Reflects all disease stages, including asymptomatic phase
U. S. (age ≥ 65 years)	≈6.9 million (2024 estimate)—Alzheimer's association report 2024	Affects ≈ 10.9% of Americans aged 65 and older
U.S. age 65–74 years	≈5.0% prevalence	Represents lower-risk age group
U.S. age ≥85 years	≈33.4% prevalence	Risk increases exponentially with age
China (≥60 years)	≈9.8 million people with AD (2020 estimate)—China CDC report	Accounts for ≈20% of global AD cases due to large aging population

### 4.2 Tau plays a role in depression

A large number of studies have shown that tau protein has an important relationship with the occurrence and development of depression Animal studies have found that mice with a mutation in the tau gene exhibit depression-related behaviors (86–88). Phosphorylation of Tau protein in the hippocampus is associated with depression (89, 90). Tau protein is a key mediator for neuronal dysfunction and related cognitive and emotional impairments after chronic stress. It is crucial for chronic stress-induced hippocampal dendrite atrophy and disruption of neuronal connections. Animals lacking Tau protein are protected from the harmful behaviors of chronic stress. Chronic stress leads to the accumulation of Tau protein and different subtypes of hyperphosphorylated Tau protein in the cytoplasm and synapses of hippocampal neurons, thereby causing depressive-like behaviors such as anhedonia in miceIn recent years (91). Wen et al. found that chronic mild unpredictable stress stimulation (CUMS) induced depressive-like behaviors in C57BL/6 mice. In the cytoplasm and synapses of hippocampal neurons in these mice, The levels of total Tau protein and hyperphosphorylated Tau protein at pSer396 and pSer404 sites increased. However, Tau KO mice did not exhibit anhedonic, anxious or depressibil-like behaviors after CUMS, meaning that after Tau protein knockout, the mice “escaped” the harmful effects caused by CUMS. Tau gene knockout mice did not exhibit any stress-induced depressive behaviors or hippocampal dysfunction (92). Khan et al. (93) discovered that hTau mice containing wild-type human MAPT transgenic exhibited depressive-like behavior at 4 months of age when their cognitive function was intact, and this depressive-like behavior was related to the increased phosphorylation of Tau protein in the dorsal nucleus. Babulal et al. (5) have found that depression is associated with tau accumulation through positron emission tomography (PET) in cognitively normal subjects. The study included 301 subjects, who underwent PET scans of their brains and analyzed the association between depression and tau and Aβ by building A logistic regression model. The results showed that patients with abnormal tau proteins were more likely to develop depression. Moriguchi et al. used PET to detect the accumulation of Tau protein and Aβ in the brains of patients with depression and healthy controls. The results indicated that Tau protein deposition might be the basis for the onset of depression (94). Gatchel et al. (95) reported that in cognitively normal older adults, depressive symptoms are associated with the accumulation of tau protein in the brain's inferior temporal lobe and entorhinal cortex. Therefore, tau-targeted therapies are also potential targets for the prevention and treatment of tau-associated depression.

The strong association between tau pathology and depressive symptoms observed in both animal models and post-mortem human studies highlights the bidirectional link between neurodegeneration and mood regulation. Experimental reduction of pathological tau alleviates depressive-like behaviors in rodents, suggesting that tau-targeted interventions may exert antidepressant effects by restoring synaptic integrity and neuronal plasticity. Translationally, positron emission tomography (PET) imaging using tau-specific tracers [e.g., (∧18F)AV-1451, (∧18F)MK-6240] has enabled the *in vivo* assessment of tau deposition in patients with late-life depression and early-stage AD, providing a bridge between preclinical findings and clinical relevance. These advances indicate that tau-based therapeutics could represent a promising translational approach for treating depression associated with neurodegenerative disorders.

## 5 Summary

Alzheimer's disease (AD) is the most prevalent tauopathy in the elderly, in which tau protein plays a central role in disease onset and progression. The pathogenesis of AD is strongly associated with abnormal tau modifications, including hyperphosphorylation, truncation, and aggregation, leading to loss of microtubule stability and “prion-like” propagation of tau pathology across connected brain regions. Soluble tau oligomers, rather than insoluble neurofibrillary tangles, are considered the most neurotoxic species, disrupting synaptic function and triggering neuronal death. Post-translational modifications such as phosphorylation, acetylation, and O-GlcNAcylation further modulate tau's aggregation propensity and toxicity.

Although β-amyloid (Aβ) deposition was long regarded as the initiating event in AD, the limited efficacy of anti-Aβ therapies has shifted research attention toward tau-centered mechanisms. tau-targeted strategies—such as inhibition of tau expression, modulation of phosphorylation, prevention of aggregation, microtubule stabilization, and active or passive immunotherapy—represent promising avenues for intervention. Most of these approaches, however, remain at the preclinical or early clinical stage, and their long-term safety and therapeutic efficacy require further validation.

Depressive symptoms are common in patients with AD and may serve both as a risk factor and a clinical manifestation of disease progression. Increasing evidence suggests that tau pathology, rather than Aβ accumulation, is closely linked to depression-related neurobiological changes. Therefore, targeting tau may not only offer potential benefits in modifying AD progression but also provide novel therapeutic opportunities for AD-associated depression.
